# Study of Gas Flow Characteristics in Tight Porous Media with a Microscale Lattice Boltzmann Model

**DOI:** 10.1038/srep32393

**Published:** 2016-09-02

**Authors:** Jianlin Zhao, Jun Yao, Min Zhang, Lei Zhang, Yongfei Yang, Hai Sun, Senyou An, Aifen Li

**Affiliations:** 1 School of Petroleum Engineering, China University of Petroleum, Qingdao, Shandong, 266580, China

## Abstract

To investigate the gas flow characteristics in tight porous media, a microscale lattice Boltzmann (LB) model with the regularization procedure is firstly adopted to simulate gas flow in three-dimensional (3D) digital rocks. A shale digital rock and a sandstone digital rock are reconstructed to study the effects of pressure, temperature and pore size on microscale gas flow. The simulation results show that because of the microscale effect in tight porous media, the apparent permeability is always higher than the intrinsic permeability, and with the decrease of pressure or pore size, or with the increase of temperature, the difference between apparent permeability and intrinsic permeability increases. In addition, the Knudsen numbers under different conditions are calculated and the results show that gas flow characteristics in the digital rocks under different Knudsen numbers are quite different. With the increase of Knudsen number, gas flow in the digital rocks becomes more uniform and the effect of heterogeneity of the porous media on gas flow decreases. Finally, two commonly used apparent permeability calculation models are evaluated by the simulation results and the Klinkenberg model shows better accuracy. In addition, a better proportionality factor in Klinkenberg model is proposed according to the simulation results.

With the development of advanced techniques such as horizontal drilling and multi-stage hydraulic fracturing, unconventional oil and gas resources are drawing more and more attention all over the world. As an important type of unconventional resource, shale gas reservoir has been explored successfully in North America^1^. However, gas flow mechanisms in such microscale pores have not been understood clearly^2^.

In recent years, with the help of advanced experimental techniques, such as scanning electron microscope (SEM), focused ion beam scanning electron microscope (FIB-SEM), micro- and nano- computed tomography (CT) etc., we can observe the detailed pore structures in shale rocks^3^. The previous studies showed that most pores in shale rocks are quite small, usually in nanoscale^3^. For gas flow in such small pores, the collisions between gas molecules and solid walls are much more obvious than those in conventional pores. This will cause the slip velocity on the solid boundaries and make gas flow in such pores very different from that in conventional pores^4^,^5^,^6^. Knudsen number (*Kn*) is the characteristic parameter for gas flow in microscale pores. It is defined as the ratio of molecular mean free path to the characteristic length of porous medium. According to Knudsen number the fluid flow can be divided into four regions^4^: continuum flow region (*Kn* < 0.001), slip flow region (0.001 < *Kn* < 0.1), transient flow region (0.1 < *Kn* < 10) and free molecular flow region (*Kn* > 10). For fluid flow in continuum flow region and slip flow region, the continuum hypothesis is valid and the Navier-Stokes (N-S) equation with non-slip boundary condition or slip boundary condition can be used to describe the flow. However, for fluid flow beyond the slip flow region, i.e. the transition flow region and free molecular flow region, the continuum hypothesis is no longer applicable, so the particle-based method should be adopted to describe the flow, such as the molecular dynamics (MD) method, direct simulation Monte Carlo (DSMC) method, lattice Boltzmann method (LBM)^7^, etc. MD^8^ is an accurate method since it describes the exact forces between molecules. However, it needs too many computation resources. So it is only suitable to simulate gas flow in quite small pores, such as nano-channels. DSMC has been successfully adopted to simulate the high Knudsen number gas flow at high speed, but it has some defects to simulate gas flow in nanopores with low speed^9^, such as the gas flow in tight porous media. In recent years, LBM has also been adopted to investigate gas flow in microscale pores^10^. Compared with MD and DSMC methods, LBM is more efficient. So it can be used to simulate gas flow in relatively large porous media. However, although LBM can be obtained by discretizing the Boltzmann equation, it omits the high order terms. So it is the approximation of the Boltzmann equation and it is not so accurate as the MD and DSMC methods. With several years’ development, LBM is considered to be accurate enough to do the microscale gas flow simulations now.

Since LBM is a N-S equation solver, it can be used to simulate gas flow in the continuum flow region by adopting the non-slip boundary condition, or in the slip flow region by adopting the slip boundary condition^11^,^12^,^13^. With the increase of Knudsen number (*Kn* > 0.1), the non-continuum effect becomes more pronounced and the N-S equation is no longer applicable. To extend LBM to simulate gas flow beyond the slip flow region, many efforts have been made, mainly including two ways. One is building the higher order LB models^14^,^15^, and the other is adopting the effective relaxation time modified by Knudsen number^16^,^17^,^18^,^19^. As the latter one is accurate without losing the advantages of traditional LB model, it is more popular. However, Suga *et al*.^20^ pointed out that this kind of LB model can be adopted to simulate gas flow in microscale channels or tubes, but it cannot be used to simulate gas flow in microscale porous media because of the inadequate symmetry of the discrete model. To overcome this defect, the regularization procedure must be considered^20^,^21^,^22^.

In recent years, LBM has been adopted to simulate gas flow in shale gas reservoirs. Fathi *et al*. firstly introduced LBM into shale gas flow simulation in 2012. They adopted the bounce back boundary condition and Langmuir slip boundary condition to investigate the Klinkenberg slip phenomenon^23^ and gas flow characteristics in inorganic and organic micro-channels^24^, respectively. However, their simulation results were questionable because the slip velocities were generated by the discretization error of their models^25^. Since then, more and more researchers adopted LBM to study gas flow characteristics in shale rocks. Zhang *et al*.^26^ adopted the microscale LB model to simulate gas flow in micro-capillary and investigated the slip effects and permeability under different Knudsen numbers. Ning *et al*.^27^ introduced the adsorption effect into the microscale LB model and adopted it to simulate shale gas flow in organic channels and two-dimensional porous media. Ren *et al*.^25^ proposed a microscale LB model considering the effects of surface diffusion, gas slippage and adsorbed layer, and adopted it to investigate the effects of surface diffusion and gas slippage on gas flow in micro-channels. According to our literature review, most of the researchers adopted the microscale LB model to investigate shale gas flow characteristics in simple channels or tubes, because the slip boundary conditions are difficult to conduct on the complex solid boundaries in porous media, especially the 3D porous media. Although some other researchers also adopted LBM to simulate gas flow in microscale porous media^28^,^29^,^30^, the geometries of their physical models are simple compared with the real rocks. In addition, they didn’t take the regularization procedure into account in their models. So their models are unsuitable for gas flow simulation in microscale porous media under relatively high Knudsen numbers. All the researches mentioned above adopted the common microscale LB model, in which the relaxation time is determined by Knudsen number and the slip boundary condition is adopted on the solid boundaries. In recent years, Chen *et al*.^31^,^32^ proposed a different LB model to consider the microscale effect based on the dusty gas model. They simulated the viscous flow and Knudsen diffusion separately with different LB models and the total mass flux can be considered as a combined result of viscous flow and Knudsen diffusion. Although this model can be used to simulate microscale gas flow in real digital rocks under any Knudsen numbers, it is not based on the kinetic theory and it could not obtain the detailed velocity distributions in the porous media. As this work mainly focuses on the gas flow characteristics in microscale porous media, the common microscale LB model is adopted here.

In this work, the common microscale LB model is firstly combined with the digital rock technology to investigate gas flow characteristics in real tight rocks. The regularization procedure is introduced into the model to enable it to simulate gas flow in porous media under high Knudsen numbers, which could not be simulated in others’ work^27^,^28^,^29^,^30^. Because of the random pore size distribution, the characteristic length is not a constant. The local characteristic length is introduced into the model which was always ignored in others’ work^27^,^28^,^29^,^30^. In addition, the diffuse reflection boundary condition is adopted to deal with the random solid boundaries. The effects of pressure, temperature and pore size on microscale gas flow are investigated firstly at the pore scale and the influencing mechanisms are analyzed. Then the gas flow characteristics in digital rocks under different Knudsen numbers are studied and some new phenomena have been found. Finally, the simulation results are adopted to verify the accuracy of two commonly used apparent permeability calculation models and a modified Klinkenberg model is proposed according to the simulation results.

## Results

The microscale LB model is adopted to simulate gas flow in digital rocks. The effects of pressure, temperature and pore size on microscale gas flow are investigated and the gas flow characteristics in tight porous media under different Knudsen numbers are analyzed.

### Digital rocks

**Figure 1:**
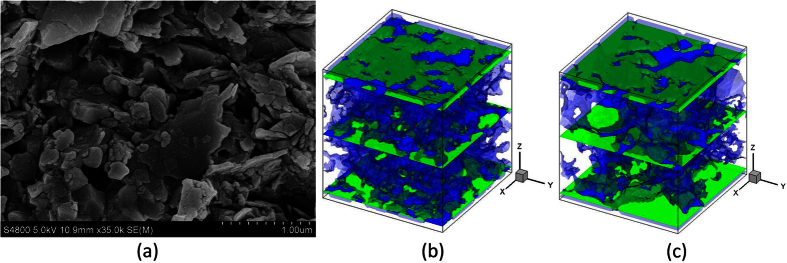
Reconstruction of the digital rocks. (**a**) SEM scanning image of a shale rock from Sichuan Basin, China. The size of the image is 1280 × 960, and the resolution is 2.825 nm per pixel. (**b**) Shale digital rock (80 × 80 × 86). (**c**) Sandstone digital rock (80 × 80 × 86). The blue part is the pore space and the green part is solid.

Two different digital rocks are used for the microscale gas flow simulations, as shown in Fig. 1(b,c). The shale digital rock is reconstructed by Markov Chain Monte Carlo (MCMC) method based on the SEM scanning image shown in Fig. 1(a) and it is adopted to study the effect of pressure and temperature on microscale gas flow. As this work mainly focuses on the microscale gas flow characteristics in tight porous media, the effect of kerogen is not taken into account here. To get the digital rock with the presence of kerogen, one can refer to the reference^33^. The sandstone digital rock is obtained by micro-CT scanning method. Although the real resolution of this digital rock is determined, by selecting different resolutions manually, we can investigate the effect of pore size on microscale gas flow. For more information about the MCMC method and micro-CT scanning method, one can refer to the references^33^,^34^.

Firstly, the traditional LB model not considering the microscale effect is adopted to simulate pressure driven gas flow in these two digital rocks. The top is inlet and the bottom is outlet. Pressure boundary condition is adopted in the *z* direction. For the *x* and *y* directions, periodic boundary condition is adopted. When the simulations reach the steady state, the intrinsic permeability and equivalent pore size of each digital rock are calculated based on the simulation results. The intrinsic permeability can be obtained based on Darcy’s law and the equivalent pore size can be obtained by the following equation:


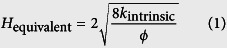


where *H*_equivalent_ is the equivalent pore size; *k*_intrinsic_ is the intrinsic permeability; *ϕ* is the porosity. The equivalent pore sizes of the two digital rocks are 2.84 and 3.55 in lattice unit, respectively. They are adopted to calculate the equivalent Knudsen numbers in the following simulations. Then pressure driven gas flow in these two digital rocks is simulated by the microscale LB model to investigate the gas flow characteristics in tight porous media.

### The effect of pressure on microscale gas flow

To investigate the effect of pressure on microscale gas flow, methane flow in the shale digital rock under different pressures is simulated by the microscale LB model. The temperature is 373 K and the inlet-outlet pressure differences are all 0.001 MPa. Although the pressures in shale gas reservoirs are always very high, the microscale effect is more obvious at low pressure. To investigate the effect of pressure on microscale gas flow more thoroughly, a wide range of pressure is adopted here. In the following simulations, the outlet pressures are 0.1 MPa, 0.2 MPa, 0.5 MPa, 1.0 MPa, 2.0 MPa, 5.0 MPa, 10.0 MPa, 20.0 MPa, 30.0 MPa, 40.0 MPa and 50.0 MPa, respectively.

**Figure 2:**
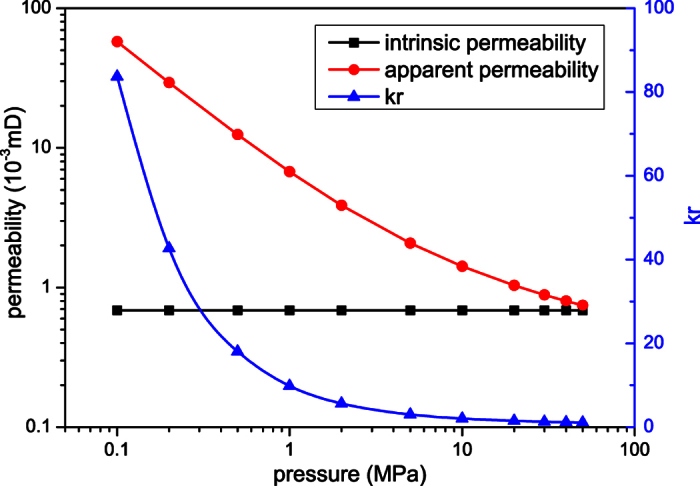
The effect of pressure on microscale gas flow.

When the simulations reach the steady state, the volume flux across the digital rock can be obtained and the apparent permeability *k*_a_ can be calculated based on Darcy’s law. The effect of pressure can be reflected by *k*_r_ which is defined as *k*_r_ = *k*_a_/*k*_intrinsic_. The simulation results are shown in Fig. 2. Pressure has a significant effect on gas flow in microscale porous media. When the pressure is very high, the apparent permeability of the shale digital rock is similar to the intrinsic permeability. With the decrease of pressure, the apparent permeability increases gradually. Especially when the pressure is very low (smaller than 1.0 MPa), the apparent permeability increases dramatically with the decrease of pressure.

**Figure 3:**
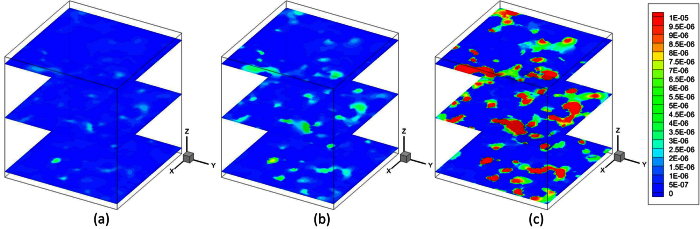
The velocity distributions in the shale digital rock under different outlet pressures. The velocity is in the lattice unit. (**a**) *P*_out_ = 50.0 MPa; (**b**) *P*_out_ = 5.0 MPa; (**c**) *P*_out_ = 0.5 MPa.

This can be explained by Fig. 3 which shows the velocity distributions in the shale digital rock under three different outlet pressures. As can be seen from Fig. 3, pressure has a significant effect on gas flow in the digital rock. When the pressure is high, gas velocity in the digital rock is very small; with the decrease of pressure, gas velocity in the digital rock increases dramatically. This is caused by the different gas flow mechanisms under different pressures. When the pressure is high, the gas molecules are close to each other. The molecular mean free path is small compared with the pore size. Therefore, the intermolecular collisions are much more frequent than the collisions between gas molecules and solid walls. There are almost no slip velocities on the solid walls. As a result, the velocity in the digital rock is small and the apparent permeability is similar to the intrinsic permeability. With the decrease of pressure, the molecular mean free path increases. The collisions between gas molecules and solid walls become increasingly obvious compared with the intermolecular collisions. Therefore, the slip velocities on the solid walls increase and the velocity in the whole digital rock increases. The apparent permeability increases accordingly.

### The effect of temperature on microscale gas flow

The shale digital rock is also adopted to investigate the effect of temperature on microscale gas flow. The outlet pressure is 20.0 MPa and the inlet pressure is 20.001 MPa. The temperatures are 298 K, 323 K, 348 K, 373 K, 398 K, 423 K, 448 K and 473 K, respectively.

**Figure 4:**
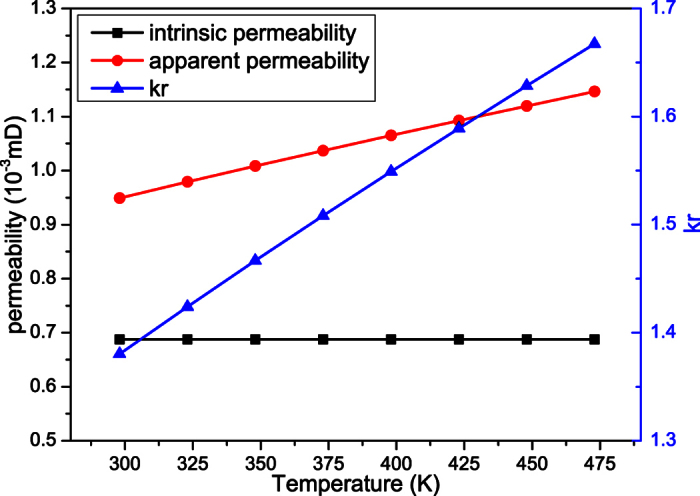
The effect of temperature on microscale gas flow.

When the simulations reach the steady state, the results can be obtained, as shown in Fig. 4. The apparent permeability increases with the increase of temperature. As the intrinsic permeability remains unchanged, *k*_r_ increases with the temperature. This is because the molecular mean free path is related to the temperature. With the increase of temperature, the kinetic energy of the gas molecules increase and they move faster. Then the collisions between gas molecules and solid walls become stronger. To keep the same pressure, the gas density must decrease. Thus the molecular mean free path increases and the collisions between gas molecules and solid walls become more obvious. Therefore, the microscale effect increases and the apparent permeability increases.

### The effect of pore size on microscale gas flow

The sandstone digital rock is adopted to investigate the effect of pore size on microscale gas flow. Methane is adopted in the simulations and the temperature is 373 K. The physical size of the digital rock can be changed by selecting different resolutions. The following resolutions are adopted here: 3.9 nm/pixel, 5.5 nm/pixel, 7.8 nm/pixel, 11.0 nm/pixel, 15.6 nm/pixel, 22.0 nm/pixel, 39.0 nm/pixel, 55.0 nm/pixel, 78.0 nm/pixel, 110.0 nm/pixel and 156.0 nm/pixel. As analyzed in above simulations, the microscale effect decreases with the increase of pressure. To magnify the effect of pore size, a relatively low pressure is adopted here. In the following simulations, the outlet pressures are all 1.0 MPa and the pressure gradients are all 6.25 × 10^−7^ MPa/nm.

**Figure 5:**
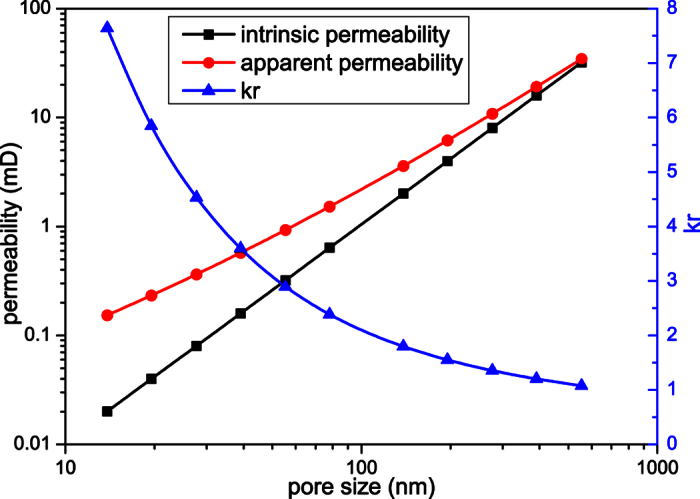
The effect of pore size on microscale gas flow.

When the simulations reach the steady state, the results can be obtained, as shown in Fig. 5. The equivalent pore size is set as the *x*-axis. As can be seen from Fig. 5, the apparent permeability is always higher than the intrinsic permeability, and with the decrease of pore size, both the intrinsic permeability and apparent permeability decrease, but *k*_r_ increases. When the pore size is large, the molecular mean free path is small compared with the characteristic length. So the microscale effect is not obvious and the apparent permeability is similar to the intrinsic permeability. As the pore size decreases, the collisions between gas molecules and solid walls become more important. So the microscale effect increases and the slip velocities on the solid walls increase. Then the difference between the apparent permeability and intrinsic permeability (*k*_r_) increases.

### Gas flow characteristics in digital rocks under different Knudsen numbers

As stated in the introduction, Knudsen number is the characteristic parameter for gas flow in microscale porous media. So the Knudsen numbers of gas flow in the digital rocks under different conditions are calculated and the corresponding gas flow characteristics are analyzed.

**Figure 6:**
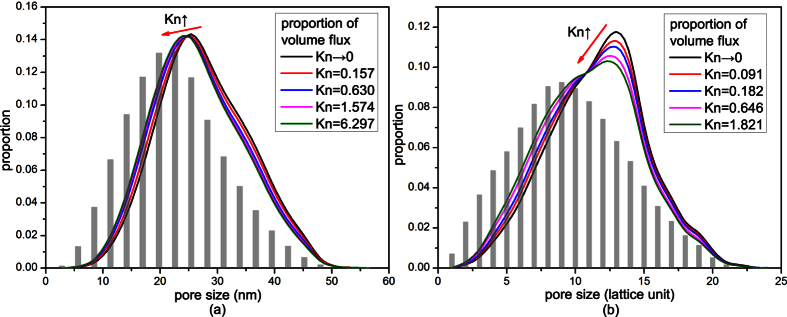
The proportions of volume flux in pores of different sizes. The histograms are the proportions of pore volume of pores with different sizes. (**a**) Shale digital rock. The curve of *Kn*→0 is obtained without considering microscale effect, while the other four curves are obtained at 373 K under the following pressures: 20.0 MPa, 5.0 MPa, 2.0 MPa and 0.5 MPa. (**b**) Sandstone digital rock. The curve of *Kn*→0 is obtained without considering microscale effect, while the other four curves are obtained under the following resolutions: 78.0 nm/pixel, 39.0 nm/pixel, 11.0 nm/pixel and 3.9 nm/pixel.

**Figure 7:**
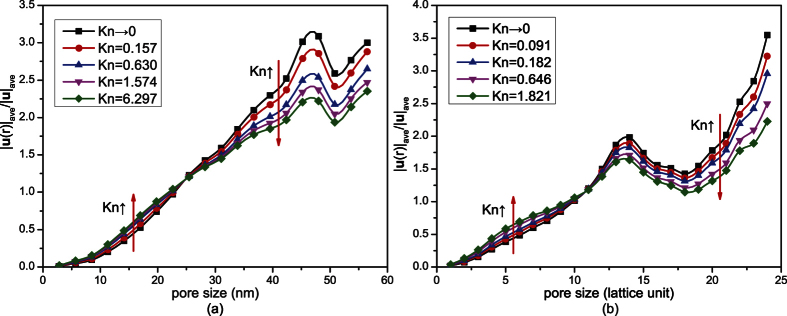
Dimensionless average velocity in pores of different sizes under different Knudsen numbers. |**u**(***r***)|_ave_ is the average gas velocity in pores with size of *r*, and |**u**|_ave_ is the average gas velocity of the whole digital rock. (**a**) Shale digital rock. (**b**) Sandstone digital rock.

Figure 6 shows the proportions of volume flux in pores of different sizes under different Knudsen numbers. The pore volume distributions are also shown in this figure. As can be seen from Fig. 6, in small pores, the proportion of pore volume is higher than that of volume flux; while in large pores, the proportion of volume flux is higher. So the velocities in small pores are smaller than those in large pores, as shown in Fig. 7. With the increase of Knudsen number, the proportion distribution curve of volume flux in Fig. 6 moves to the lower left. So the relative volume fluxes in small pores increase while those in large pores decrease. As a result, the dimensionless average velocities in small pores increase while those in large pores decrease, as shown in Fig. 7. This means that the difference of gas flow resistances in pores of different sizes decreases. So gas flow in the digital rocks becomes more uniform and the effect of heterogeneity of the porous media on gas flow decreases.

The following statements can explain the above phenomena. Gas flow in the porous media is motivated by the intermolecular collisions and collisions between gas molecules and solid walls. When Knudsen number is very small, gas flow is mainly dominated by the intermolecular collisions. There exist very small slip velocities on the solid walls in both small pores and large pores. In large pores there are more intermolecular collisions, while in small pores there are less intermolecular collisions. So the gas flow ability in large pores is much higher than that in small pores. The proportion of volume flux and dimensionless velocity in large pores are higher and the effect of heterogeneity of the porous media is obvious. With the increase of Knudsen number, the effect of the collisions between gas molecules and solid walls becomes increasingly obvious, so the slip velocities on the solid walls increase and the gas flow resistance decreases. In addition, the Knudsen numbers in small pores are higher than those in large pores because of their smaller sizes. So the microscale effect is more obvious and the gas flow resistance decreases more in small pores. Therefore, the difference of gas flow abilities in large pores and small pores decreases. Then gas flow in the whole digital rock becomes more uniform and the effect of heterogeneity of the porous media on gas flow decreases.

### Evaluation of two apparent permeability calculation models

According to the simulation results, in tight porous media, such as shale gas reservoirs, because of the microscale effect caused by the ultra small sizes of pores, the permeability is no longer a constant. The apparent permeability is always used in macroscale shale gas reservoir simulations. Klinkenberg model^35^ and Beskok-Karniadakis model (B-K model)^36^ are two commonly used apparent permeability calculation models. For 3D fluid flow in porous media, they have the following formats.

Klinkenberg model:


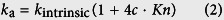


where *c* is the proportionality factor. Klinkenberg pointed out that the value of *c* seems to be slightly less than 1.0^35^. However for most researchers, they usually choose *c* = 1.0^37^. In this work, we also choose *c* = 0.8 for comparison.

B-K model:


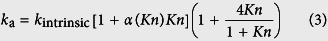


where


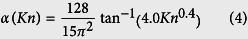


**Figure 8:**
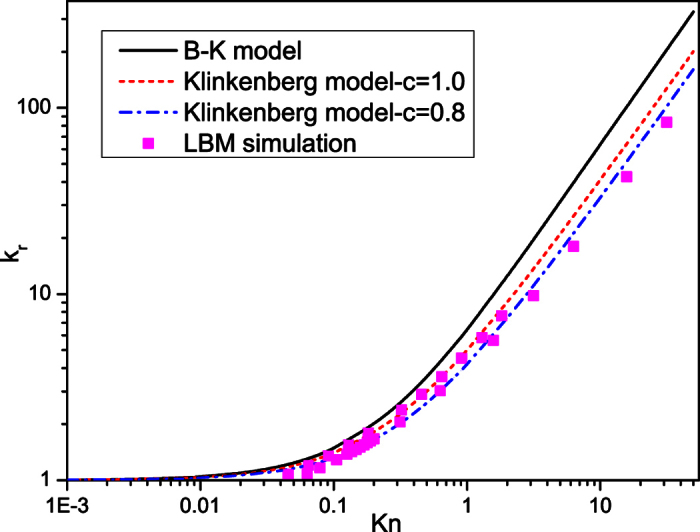
Comparison of the calculated results of two apparent permeability calculation models with the simulation results of LBM.

LBM is adopted to evaluate the accuracy of these two models. Figure 8 shows the comparison of the calculated results of the two models with the simulation results of LBM. As shown in Fig. 8, when the Knudsen number is small, *k*_r_ increases slowly with the increase of Knudsen number. Both models can well describe the microscale effect. But when Knudsen number gets higher, *k*_r_ increases quickly as Knudsen number increases. The B-K model will obviously overestimate the microscale effect, while the Klinkenberg model gives a better prediction. In addition, our simulation results suggest that the proportionality factor *c* in Klinkenberg model should be modified to 0.8.

After getting the modified Klinkenberg model, our simulation results can be used in the following way. For any porous medium, it can be equivalent to the capillary bundles. Then the equivalent pore size can be obtained according to Eq. (1), and the corresponding Knudsen number can be calculated by Eq. (14). After obtaining the Knudsen number, the modified Klinkenberg model can be used to calculate the apparent permeability of the tight porous medium, which is a critical parameter in the macroscale numerical simulation of tight gas or shale gas reservoirs.

## Discussion

In this work, a microscale LB model with the regularization procedure is adopted to simulate gas flow in 3D digital rocks. The diffuse reflection boundary condition is adopted to deal with the random solid boundaries and the local characteristic lengths of pores with different sizes are also introduced into the model. A shale digital rock and a sandstone digital rock are adopted to investigate the gas flow characteristics in microscale porous media. Gas flow simulations in the shale digital rock under different pressures and temperatures are conducted to investigate the effects of pressure and temperature on microscale gas flow. And the sandstone digital rock is used to study the effect of pore size on microscale gas flow by selecting different resolutions. The simulation results show that with the decrease of pressure or pore size, or with the increase of temperature, the difference between apparent permeability and intrinsic permeability increases. The decrease of pressure and the increase of temperature will increase the molecular mean free path while the decrease of pore size will decrease the characteristic length, all will make the collisions between gas molecules and solid walls become more obvious compared with the intermolecular collisions. As a result, the slip velocities on the solid walls increase and the apparent permeability increases. In addition, the results show that gas flow characteristics in the digital rocks under different Knudsen numbers are quite different. With the increase of Knudsen number, the dimensionless average velocities in small pores increase while those in large pores decrease. Gas flow in the digital rocks becomes more uniform and the effect of heterogeneity of the porous media on gas flow decreases. Finally, the Klinkenberg model and B-K model for calculating the apparent permeability of tight porous media are evaluated by the LBM simulation results. According to the simulation results, we suggest the Klinkenberg model in macroscale shale gas reservoir simulations because of its better accuracy. In addition, a better proportionality factor (*c* = 0.8) in Klinkenberg model is proposed according to the simulation results.

Kerogen is very common and important in shale gas reservoirs. However, this work mainly focuses on the gas flow characteristics in tight porous media. In our future work, we will investigate the adsorption effect of kerogen on microscale gas flow.

## Methods

### Lattice Boltzmann method

In this work, the 3D microscale LB model with the D3Q19 discrete velocity model^38^ is adopted to simulate gas flow in digital rocks. The basic evolution equation with the Bhatnagar-Gross-Krook (BGK) collision approximation is shown as follows. Unless otherwise stated, all units in this paper are lattice units.


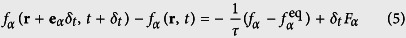


where *f*_*α*_ is the density distribution function of *α* direction; *α* = 0, 1, 2, …, 18; **r** is the spatial location of the particles; **e**_*α*_ is the velocity of *α* direction; *t* is time; *δ*_*t*_ is time step; *τ* is the relaxation time; *F*_*α*_ is the force term; 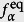 is the local equilibrium distribution function of *α* direction:


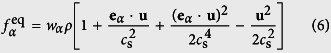


where *ρ* and **u** are the macroscopic density and velocity, respectively; *c*_s_ is the lattice sound speed; *w*_*α*_ is the weight factor of *α* direction.

The force term in Eq. (5) can be obtained by Hermite expansion. For the D3Q19 model, it can be expressed as^20^:


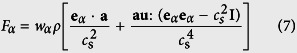


where **a** is the acceleration of the force.

### Relaxation time and boundary condition

For gas flow in microscale pores, the relaxation time should be determined by Knudsen number. Considering the microscale effect and the effect of Knudsen layer, the relaxation time can be expressed as^19^:


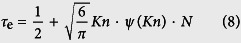


where *τ*_e_ is the effective relaxation time considering the effect of Knudsen layer; *ψ*(*Kn*) is the modification function and we set *ψ*(*Kn*) = 1/(1 + 2*Kn*)^19^; *N* = H/Δ*x* means the number of lattices occupied by the characteristic length, where *H* is the characteristic length, m, and Δ*x* is the size of one lattice, m.

As the solid boundaries in shale are quite rough, the diffuse reflection boundary condition is very appropriate for such boundaries. The discrete format of the diffuse reflection boundary condition in LBM is^11^,^13^:


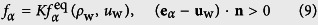


where


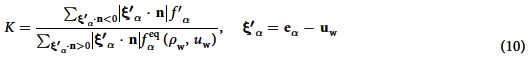


where **n** is the inward unit normal vector; **u**_w_ is the wall velocity; the subscript w means the solid walls; 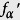 is the distribution function after streaming. As long as the inward normal direction can be obtained, the diffuse reflection boundary condition can be adopted.

### Regularization procedure

To apply the microscale LB model into gas flow simulation in porous media, the regularization procedure must be introduced^20^,^39^. With the regularization procedure, the evolution equation becomes:


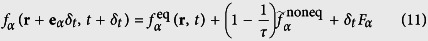


where 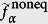 is the regularized distribution function and can be expressed as:


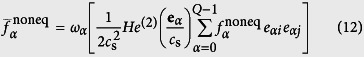


where 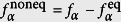 is the non-equilibrium part of the distribution function. *He*^(2)^ is the second Hermite polynomial^21^. For more information about the regularization procedure one can refer to the references^20^,^39^.

### Local Knudsen number

According to the kinetic theory, for hard sphere molecules, the molecular mean free path is^16^:


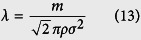


where *m* is the molecular weight and *m* = 2.658 × 10^−26^ kg for methane; *σ* is the molecular diameter and *σ* = 3.8 × 10^−10^ m for methane; *ρ* is gas density, kg/m^3^. According to the definition of Knudsen number, it can be expressed as:


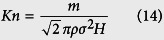


For gas flow in porous media, the characteristic length is the pore size. Because of the non-uniform pore size distribution, *H* is not a constant in porous media. The pore sizes in different positions can be obtained by the method in reference^40^. For a certain type of gas, *m* and *σ* are constants. As *ρ* is determined by the pressure, Knudsen number is related to *P* and *H*. Before the simulation, the reference characteristic length *H*_ref_ and the reference pressure *P*_ref_ should be chosen to calculate the reference Knudsen number *Kn*_ref_. Then the local Knudsen number *Kn*(**r**) at any time step can be obtained as follows.


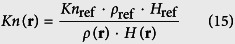


### Model verification

**Figure 9:**
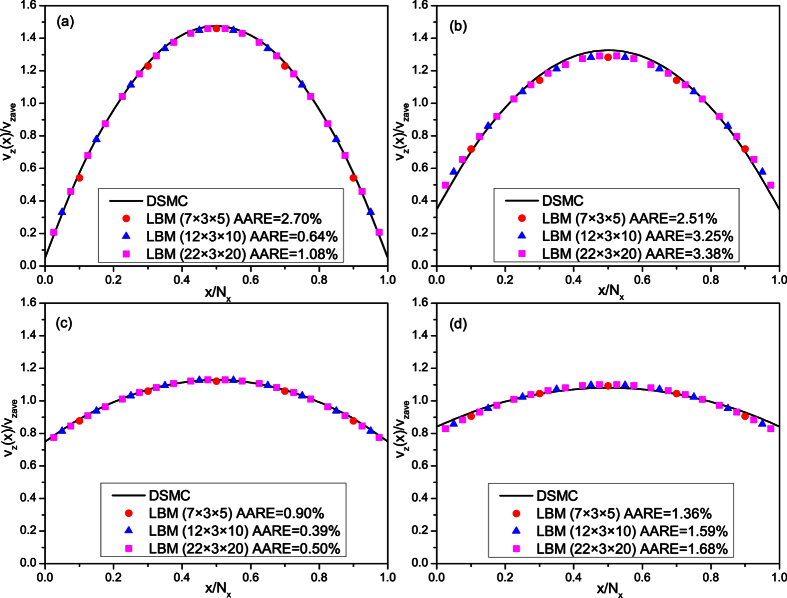
Comparison of the dimensionless streamwise velocity profiles of different methods under different *Kn*. *v*_zave_ is the average velocity in the *z* direction. AARE is the absolute average relative error. The results of LBM are obtained at *z* = *N*_*z*_/2 in slice *y* = 2. (**a**) *Kn* = 0.01; (**b**) *Kn* = 0.1; (**c**) *Kn* = 1.0; (**d**) *Kn* = 10.0.

As the nanoscale physical experiments are extremely difficult to conduct, we compare the simulation results of this model with those of MD and DSMC methods to verify the accuracy of this model. Firstly, gas flow in nano-channels under different Knudsen numbers are simulated by this model and the simulation results are compared with those of DSMC method. The physical model is a parallel slab model. To study the effect of grid refinement, three slab models with different grid numbers are adopted here: slab model 1 with *N*_*x*_ × *N*_*y*_ × *N*_*z*_ = 7 × 3 × 5, slab model 2 with *N*_*x*_ × *N*_*y*_ × *N*_*z*_ = 12 × 3 × 10 and slab model 3 with *N*_*x*_ × *N*_*y*_ × *N*_*z*_ = 22 × 3 × 20. The left and right nodes are solid nodes and the nodes in the middle area are pore nodes. Periodic boundary condition is adopted in *y* and *z* directions and a uniform pressure gradient of 5 × 10^−5^ is applied in *z* direction. Gas flow in the slab models under four different Knudsen numbers are simulated by our model and the simulation results are compared with those of DSMC method^20^, as shown in Fig. 9. The results of the LB model match well with those of DSMC method, which verifies the accuracy of this model to simulate gas flow in different flow regions. In addition, the simulation results under different grid refinement almost fall on the same line, which demonstrates that the simulation results of this LB model is independent of grid refinement.

**Figure 10:**
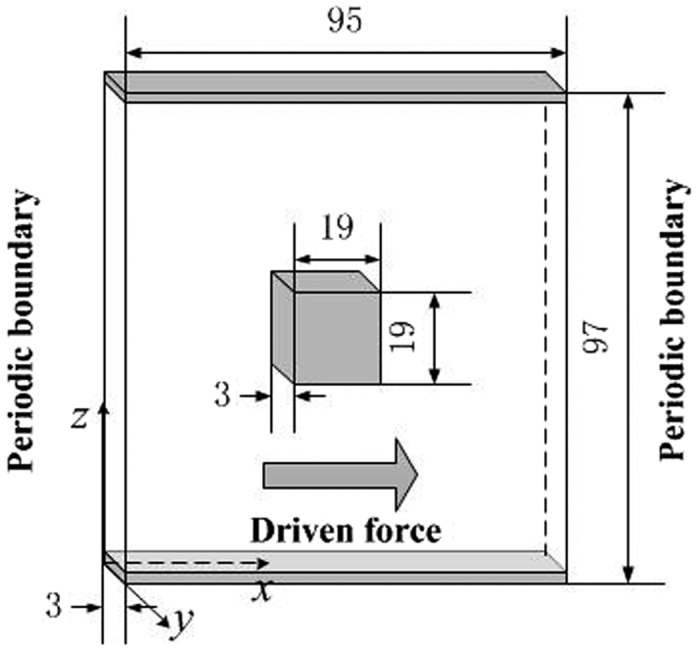
Physical model of the gas flow around a square cylinder in a nanochannel. The grey color means solids and the white color means pore space.

**Figure 11:**
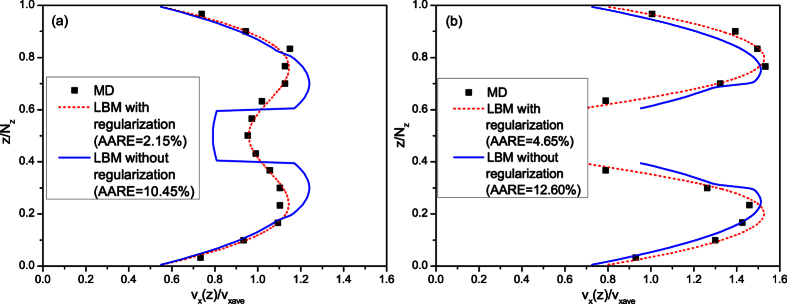
Comparison of streamwise velocity profiles in square cylinder channel flows at *Kn* = 0.11. The results of LBM are obtained at slice *y* = 2. (**a**) *x/N*_*x*_ = 0.0, (**b**) *x/N*_*x*_ = 0.5.

To further verify the accuracy of this model to simulate gas flow in microscale porous media, force driven gas flow in microscale porous media is simulated by this model and the simulation results are compared with those of MD method^41^. In addition, the microscale LB model not considering the regularization procedure is also adopted to do the same simulation. The physical model is a parallel slab model with a square cylinder in the middle^20^,^41^, as shown in Fig. 10. The resolution is 1.0 nm/pixel and methane is adopted in the simulation. The temperature is 373 K and the pressure is 2.413 MPa. Then the corresponding Knudsen number is 0.11. The driven acceleration is 5 × 10^−5^ in *x* direction. The simulation results are shown in Fig. 11. The results of the LB model with regularization procedure match well with those of MD method, but the LB model without regularization procedure will generate unphysical velocity distributions. This demonstrates that for LBM, the regularization procedure is essential for gas flow simulation in microscale porous media. As the models adopted in others’ research^27^,^28^,^29^,^30^ did not take the regularization procedure into account, they could not be adopted to simulate gas flow in porous media under relatively high Knudsen numbers.

## Additional Information

**How to cite this article**: Zhao, J. *et al*. Study of Gas Flow Characteristics in Tight Porous Media with a Microscale Lattice Boltzmann Model. *Sci. Rep*. **6**, 32393; doi: 10.1038/srep32393 (2016).

## References

[b1] Kuuskraa V., Stevens S. H. & Moodhe K. D. Technically recoverable shale oil and shale gas resources: an assessment of 137 shale formations in 41 countries outside the United States (2013).

[b2] Yao J. . Key mechanical problems in the developmentof shale gas reservoirs. Sci. Sin. Phys. Mech. Astron 43, 1527–1547 (2013).

[b3] Loucks R. G., Reed R. M., Ruppel S. C. & Hammes U. Spectrum of pore types and networks in mudrocks and a descriptive classification for matrix-related mudrock pores. AAPG bulletin 96, 1071–1098 (2012).

[b4] Yao J., Sun H., Fan D.-y., Wang C.-c. & Sun Z.-x. Numerical simulation of gas transport mechanisms in tight shale gas reservoirs. Petroleum Science 10, 528–537 (2013).

[b5] Sun H., Yao J., Fan D.-y., Wang C.-c. & Sun Z.-x. Gas transport mode criteria in ultra-tight porous media. International Journal of Heat and Mass Transfer 83, 192–199 (2015).

[b6] Zhang M. . Triple-continuum modeling of shale gas reservoirs considering the effect of kerogen. Journal of Natural Gas Science and Engineering 24, 252–263 (2015).

[b7] Chen L., Luan H.-B., He Y.-L. & Tao W.-Q. Pore-scale flow and mass transport in gas diffusion layer of proton exchange membrane fuel cell with interdigitated flow fields. International Journal of Thermal Sciences 51, 132–144 (2012).

[b8] Bird G. A. Molecular gas dynamics and the direct simulation of gas flows (1994).

[b9] Shen C. Rarefied gas dynamics: fundamentals, simulations and micro flows. (Springer Science & Business Media, 2006).

[b10] Nie X., Doolen G. D. & Chen S. Lattice-Boltzmann simulations of fluid flows in MEMS. Journal of Statistical Physics 107, 279–289 (2002).

[b11] Ansumali S. & Karlin I. V. Kinetic boundary conditions in the lattice Boltzmann method. Physical Review E 66, 026311 (2002). 10.1103/PhysRevE.66.026311 12241289

[b12] Succi S. Mesoscopic modeling of slip motion at fluid-solid interfaces with heterogeneous catalysis. Physical review letters 89, 064502 (2002). 12190587 10.1103/PhysRevLett.89.064502

[b13] Tang G., Tao W. & He Y. Lattice Boltzmann method for gaseous microflows using kinetic theory boundary conditions. Physics of Fluids (1994-present) 17, 058101 (2005).

[b14] Shan X., Yuan X.-F. & Chen H. Kinetic theory representation of hydrodynamics: a way beyond the Navier-Stokes equation. Journal of Fluid Mechanics 550, 413–441 (2006).

[b15] Ansumali S., Karlin I., Arcidiacono S., Abbas A. & Prasianakis N. Hydrodynamics beyond Navier-Stokes: Exact solution to the lattice Boltzmann hierarchy. Physical review letters 98, 124502 (2007). 17501130 10.1103/PhysRevLett.98.124502

[b16] Guo Z., Zhao T. & Shi Y. Physical symmetry, spatial accuracy, and relaxation time of the lattice Boltzmann equation for microgas flows. Journal of Applied physics 99, 074903 (2006).

[b17] Zhang Y.-H., Gu X.-J., Barber R. W. & Emerson D. R. Capturing Knudsen layer phenomena using a lattice Boltzmann model. Physical Review E 74, 046704 (2006). 10.1103/PhysRevE.74.046704 17155209

[b18] Guo Z., Zheng C. & Shi B. Lattice Boltzmann equation with multiple effective relaxation times for gaseous microscale flow. Physical Review E 77, 036707 (2008). 10.1103/PhysRevE.77.036707 18517557

[b19] Li Q., He Y., Tang G. & Tao W. Lattice Boltzmann modeling of microchannel flows in the transition flow regime. Microfluidics and nanofluidics 10, 607–618 (2011).

[b20] Suga K. . Evaluation of a lattice Boltzmann method in a complex nanoflow. Physical Review E 82, 016701 (2010). 10.1103/PhysRevE.82.016701 20866755

[b21] Zhang R., Shan X. & Chen H. Efficient kinetic method for fluid simulation beyond the Navier-Stokes equation. Physical Review E 74, 046703 (2006). 10.1103/PhysRevE.74.046703 17155208

[b22] Niu X.-D., Hyodo S.-A., Munekata T. & Suga K. Kinetic lattice Boltzmann method for microscale gas flows: Issues on boundary condition, relaxation time, and regularization. Physical review E 76, 036711 (2007). 10.1103/PhysRevE.76.036711 17930365

[b23] Fathi E., Tinni A. & Akkutlu I. Y. Correction to Klinkenberg slip theory for gas flow in nano-capillaries. International Journal of Coal Geology 103, 51–59 (2012).

[b24] Fathi E. & Akkutlu I. Y. Lattice Boltzmann method for simulation of shale gas transport in kerogen. SPE Journal 18, 27–37 (2012).

[b25] Ren J., Guo P., Guo Z. & Wang Z. A lattice Boltzmann model for simulating gas flow in kerogen pores. Transport in Porous Media 106, 285–301 (2015).

[b26] Zhang X., Xiao L., Shan X. & Guo L. Lattice Boltzmann simulation of shale gas transport in organic nano-pores. Scientific Reports 4 (2014). 10.1038/srep04843 PMC4007072 24784022

[b27] Ning Y., Jiang Y. & Qin G. In *International Petroleum Technology Conference*. (International Petroleum Technology Conference).

[b28] Chai Z., Shi B., Guo Z. & Lu J. Gas flow through square arrays of circular cylinders with Klinkenberg effect: a lattice Boltzmann study. Communications in Computational Physics 8, 1052 (2010).

[b29] Tang G., Tao W. & He Y. Gas slippage effect on microscale porous flow using the lattice Boltzmann method. Physical Review E 72, 056301 (2005). 10.1103/PhysRevE.72.056301 16383739

[b30] Tang G., Tao W. & He Y. Three-dimensional lattice Boltzmann model for gaseous flow in rectangular microducts and microscale porous media. Journal of Applied Physics 97, 104918 (2005).

[b31] Chen L., Kang Q., Pawar R., He Y.-L. & Tao W.-Q. Pore-scale prediction of transport properties in reconstructed nanostructures of organic matter in shales. Fuel 158, 650–658 (2015).

[b32] Chen L. . Nanoscale simulation of shale transport properties using the lattice Boltzmann method: permeability and diffusivity. Scientific Reports 5 (2015). 10.1038/srep08089 PMC4308705 25627247

[b33] Yang Y. . New pore space characterization method of shale matrix formation by considering organic and inorganic pores. Journal of Natural Gas Science and Engineering 27, 496–503 (2015).

[b34] Zhang L. . Pore scale simulation of liquid and gas two-phase flow based on digital core technology. Science China Technological Sciences 58, 1375–1384 (2015).

[b35] Klinkenberg L. In Drilling and production practice. (American Petroleum Institute) (1941).

[b36] Beskok A. & Karniadakis G. E. Report: a model for flows in channels, pipes, and ducts at micro and nano scales. Microscale Thermophysical Engineering 3, 43–77 (1999).

[b37] Freeman C., Moridis G. & Blasingame T. A numerical study of microscale flow behavior in tight gas and shale gas reservoir systems. Transport in porous media 90, 253–268 (2011).

[b38] Qian Y., d’Humières D. & Lallemand P. Lattice BGK models for Navier-Stokes equation. EPL (Europhysics Letters) 17, 479 (1992).

[b39] Niu X., Hyodo S.-A., Munekata T. & Suga K. Kinetic lattice Boltzmann method for microscale gas flows: issues on boundary condition, relaxation time, and regularization. Physical Review E 76, 036711 (2007). 10.1103/PhysRevE.76.036711 17930365

[b40] Lange K. J., Sui P.-C. & Djilali N. Pore scale simulation of transport and electrochemical reactions in reconstructed PEMFC catalyst layers. Journal of the Electrochemical Society 157, B1434–B1442 (2010).

[b41] Suga K. Lattice Boltzmann methods for complex micro-flows: applicability and limitations for practical applications. Fluid Dynamics Research 45, 034501 (2013).

